# High-definition Transcranial Direct Current Stimulation Improves Cognitive Function in Patients with Cerebral Small Vessel Disease: An Open-label Pilot Study Informed by Cross-sectional Analysis

**DOI:** 10.2174/011570159X421474260311080532

**Published:** 2026-05-15

**Authors:** Shanshan Cao, Baogen Du, Yuanyuan Liu, Shihao Ruan, Wenchen Sun, Ying Zhang, Jinying Yang, Panpan Hu, Kai Wang, Qiang Wei

**Affiliations:** 1 Department of Neurology, The First Affiliated Hospital of Anhui Medical University, Hefei, 230022, China;; 2 Anhui Province Key Laboratory of Cognition and Neuropsychiatric Disorders, Hefei, 230032, China;; 3 Collaborative Innovation Center of Neuropsychiatric Disorders and Mental Health, Anhui Province, Hefei, 230032, China;; 4 Department of Psychiatry, Wuhan Wudong Hospital, Wuhan, 430084, China;; 5 Laboratory Center for Information Science, University of Science and Technology of China, Hefei, 230026, China;; 6 The School of Mental Health and Psychological Sciences, Anhui Medical University, Hefei, 230032, China;; 7 Institute of Artificial Intelligence, Hefei Comprehensive National Science Center, Hefei, 230088, China

**Keywords:** Cerebral small vessel disease, white matter hyperintensities, transcranial direct current stimulation, cognitive impairment, hippocampal subregion, magnetic resonance imaging

## Abstract

**Introduction:**

Cerebral Small Vessel Disease (CSVD) is one of the most common causes of vascular cognitive impairment and dementia, and there is still a lack of effective treatment options. High-Definition Transcranial Direct Current Stimulation (HD-tDCS) is a non-invasive neural regulation method with promising prospects in the treatment of cognitive impairment. Therefore, we explore whether HD-tDCS can improve cognitive function and potential mechanisms in patients with CSVD.

**Methods:**

This study was divided into two complementary parts. Part 1 involved 104 patients with CSVD and 36 matched Healthy Controls (HCs) to establish the optimal HD-tDCS stimulation target. Part 2 enrolled an additional 20 patients with CSVD who received a 2-week HD-tDCS intervention targeting the stimulation site identified in Part 1. Cognitive assessments and multimodal magnetic resonance imaging data were collected from all participants at both baseline and follow-up.

**Results:**

In part 1 of the study, the patients with CSVD showed significant differences in Montreal Cognitive Assessment (MoCA), Auditory Verbal Learning Test (AVLT)-study, AVLT-immediate, AVLT-delay, AVLT-recognition, Trail Making Test (TMT)-A, TMT-B, and Verbal Fluency Test -fruits/vegetables compared with HCs. Meanwhile, left hippocampal subregions to whole-brain resting state-functional connectivity (rs-FC) analysis showed that the rs-FC strength between the hippocampal cognitive subregion (HIPc) and both the left dorsolateral superior frontal gyrus and the right medial superior frontal gyrus significantly altered in patients with CSVD compared with HCs, suggesting that the prefrontal cortex is a stimulation target of HD-tDCS. In part 2 of the study, performance on the MoCA, AVLT-study, AVLT-delay, SDMT, and digit span test improved in 20 patients with CSVD after 14 days of HD-tDCS targeting the left dorsolateral prefrontal cortex. At the same time, the rs-FC strength between HIPc and the alteration cluster found in the first part of the study, located in the left dorsolateral superior frontal gyrus, was decreased after treatment.

**Discussion:**

HD-tDCS may help normalize brain network function and improve cognitive function in patients with CSVD by reducing rs-FC between the hippocampus and the left dorsolateral prefrontal cortex. Further research is needed to refine treatment plans and evaluate the long-term effects of HD-tDCS on cognitive function in patients with CSVD.

**Conclusion:**

The patients with CSVD exhibited altered rs-FC in the hippocampal-prefrontal circuit. HD-tDCS could modulate this circuit and improve cognitive function in patients with CSVD.

## 1 INTRODUCTION

Cerebral Small Vessel Disease (CSVD) is a disorder of the brain’s small perforating arterioles, capillaries, and probably venules [1], which is one of the most common cause of Vascular Cognitive Impairment (VCI) and dementia [2, 3], includes lacunes, White Matter Hyperintensities (WMHs), cerebral microbleeds (CMBs), and enlarged perivascular spaces (PVSs) [4, 5]. Large numbers of studies have investigated CSVD and incident dementia, including European and North American populations, as well as in East Asia [6-9]. Cognitive impairment in patients with CSVD may affect the entire range of cognitive functions; executive and memory dysfunction are the striking features [10]. The economic impact of cognitive impairment is enormous, and individuals with cognitive impairment will take up more health care services and require more daily life event support [11, 12].

To date, the underlying mechanisms of cognitive impairment in CSVD are still poorly understood. Therefore, effective treatment options for CSVD-related cognitive impairment are still lacking. Preventing vascular risk factors is a priority in clinical interventions for VCI, including CSVD [13, 14]. However, these methods mainly focus on improving vascular lesions and cannot effectively reverse or alleviate brain structural damage and cognitive dysfunction caused by CSVD. In addition, conventional anti dementia drugs have not yet been recommended for the treatment of VCI due to the lack of specific drug therapy for CSVD [15, 16].

Research has shown that CSVD lesions can destroy white matter fibers and their connections, affect the cholinergic system and related hormone secretion, and damage fiber connecting bundles and neurotransmission pathways, leading to information transport disruption and cognitive dysfunction. Meanwhile, cross-sectional and longitudinal studies have demonstrated that the underlying mechanisms related to CSVD are highly correlated with hippocampal subfield degeneration [17, 18]. The hippocampus plays an important role in memory and executive function, the main impaired cognitive domains of CSVD [15, 19, 20], through its connection with the prefrontal cortex [21, 22]. Meanwhile, the advances in human connectomics have led to a better understanding of how CSVD might give rise to clinical symptoms through its effects on functional connected brain networks [1]. Functional connectivity is defined as the temporal dependency of neuronal activation patterns in anatomically separated brain regions [23], and functional neuroimaging studies have reported reduced functional connectivity across distributed networks in CSVD [1], which may be an important cause of cognitive impairment in patients with CSVD.

Transcranial direct current stimulation (tDCS) is a noninvasive neuromodulation method widely used by neuroscientists and clinicians for therapeutic purposes [24], which can induce acute and sustained neuroplastic changes in cortical excitability, exerting the ability to regulate brain function [25]. Previous studies have shown that tDCS has demonstrated significant therapeutic effects in depression [26-28], epilepsy [29, 30], schizophrenia [31, 32], and neurodegenerative diseases [33-36]. The effect of conventional tDCS is essentially diffuse or unfocused. Considering the drawback of conventional tDCS, researchers have used circular electrodes to apply direct current more concentratedly to different brain regions, adjusting the cortical field and focal area stimulation density, known as High-definition tDCS (HD-tDCS), specifically, this technology injects weak direct current through scalp electrodes, and the current partially penetrates the brain, increasing or decreasing the excitability of neurons near the anode or cathode, respectively [32, 37]. The potential of HD-tDCS to enhance cognitive function has been confirmed in Alzheimer's disease patients [36, 38]. Furthermore, the left dorsolateral prefrontal cortex (DLPFC) has become the optimal intervention target for cognitive impairment related to CSVD due to its neuroplasticity and core role in executive function and memory [35, 39-42], which are mainly affected in CSVD.

Therefore, in the first part of this study, the left hippocampus was used as the region of interest for resting state functional connectivity analysis to explore the possible mechanisms of CSVD cognitive impairment and determine the optimal cortical stimulation targets for HD-tDCS therapy. The second part is to conduct an open-label pilot study based on the stimulation targets identified in the first part, to explore whether HD-tDCS can improve cognitive function and potential mechanisms in patients with CSVD.

## 2 MATERIALS AND METHODS

This study has been reviewed and approved by the Ethics Committee of Anhui Medical University (Approval Number: 2021H003). This study consists of two parts: Part 1 recruited participants to determine the cortical targets for treatment through functional connectivity analysis. Part 2 conducted a 14-day HD-tDCS treatment based on the targets identified in the part 1. The specific research process is shown in Fig. (**1**).

### 2.1 Participants

Firstly, this study recruited 104 patients with CSVD and 36 Healthy Controls (HCs) from the First Affiliated Hospital of Anhui Medical University from 2017 to 2019 to determine the parameters of HD-tDCS. The inclusion criteria were as follows: (1) age 40-80 years; (2) visible WMHs on T2 fluid-attenuated inversion recovery (T2 FLAIR) imaging. The exclusion criteria were as follows: (1) intracranial and extracranial stenosis > 50%, (2) Trial of Org 10172 in Acute Stroke Treatment classification suggestive of cardiogenic stroke, (3) non-CSVD-related WMHs (*e.g*., multiple sclerosis); (4) mental disorders or alcohol addiction, (5) dementia or tumours, (6) intracranial hemorrhage, (7) significant hearing or visual impairments or physical movement disorders that prevented co-operation during cognitive testing, (8) language barriers, and (9) Magnetic Resonance Imaging (MRI) contraindications or known claustrophobia.

HCs were relatives of patients with WMHs, along with other social recruits, and were enrolled. These controls were studied during the same period and matched the demographic data of the patients with WMHs in factors including age, gender, and years of education, and had no previous history of neurological diseases or mental illnesses. Imaging showed no WMHs in these participants.

In addition, 20 subjects were recruited for HD-TDCS treatment at the First Affiliated Hospital of Anhui Medical University from 2019 to 2021, with specific inclusion and exclusion criteria as follows: (1) age 40-80 years; (2) multiple (≥3) supratentorial subcortical small infarcts (3-20 mm in diameter), with/without WMHs of any degree; or moderate to severe WMHs (score ≥2 according to the Fazekas rating scale) with/without small infarct; or one or more strategically located subcortical small infarcts in the caudate nucleus, globus pallidus, or thalamus; (3) neither normal nor demented according to the criteria of the "Diagnostic and Statistical Manual of Mental Disorders-5th Edition, DSM-V" with clinical dementia rating ≥0.5 on at least one domain and global score ≥0.5; (4) no hippocampal or entorhinal cortex atrophy (score of zero or one according to the medial temporal lobe atrophy scale of Scheltens; (5) if a patient has a clear history of stroke, the patient would be recruited 3 months after the last stroke. The exclusion criteria were as follows: (1) intracranial and extracranial stenosis >50%; (2) Trial of Org 10172 in Acute Stroke Treatment classification suggestive of cardiogenic stroke; (3) non-CSVD-related WMHs; (4) mental disorders or alcohol addiction; (5) tumors; (6) intracranial hemorrhage; (7) significant hearing or visual impairments or physical movement disorders that prevented co-operation during neuropsychological tests; (8) language barriers; (9) MRI contraindications or known claustrophobia.

### 2.2 Determination of HD-tDCS Parameters

The position of the central electrode is an important parameter of HD-tDCS. Meanwhile, given the core role of the hippocampus in cognitive function, the first part of the study used resting-state functional connectivity analysis with hippocampal subregions [43] as seed points in patients with CSVD and Healthy Controls (HCs) to determine the position of the central electrode. Specifically, the hippocampal subregion includes the hippocampal emotional subregion (HIPe), the hippocampal cognitive subregion (HIPc), and the hippocampal perceptual subregion (HIPp). This study locates seed points in these three subregions of the left hippocampus. Pearson correlations are computed between the time series of each hippocampal subregion and each voxel across the whole brain. To normalize the correlation coefficients, Fisher’s z-transform was applied, and the functional connectivity maps between each subregion of the left hippocampus and the whole brain were obtained for each subject. The results are then visualized using the GRETNA (Graph-theoretical Network Analysis) software [44] (https://github.com/sandywang/GRETNA).

### 2.3 Treatment of HD-tDCS

The patients with CSVD underwent 14 sessions for 2 weeks. This study used the HD-tDCS instrument developed by Soterix Medical Inc. Based on the results of the first part, considering that the position of the right medial superior frontal gyrus is too deep for HD-tDCS to accurately stimulate, the stimulation electrode (central electrode) was placed on the left DLPFC and based on the standard EEG 10-20 EEG localization system, it was placed at F3. The other four peripheral electrodes were placed at Fp1, Fz, C3, and F7. The current intensity is set to 2 milliamps, and the stimulation time is set to 20 minutes (including a 30-second current rise and fall to prevent discomfort symptoms such as stinging caused by rapid current rise or fall). The subjects received HD-tDCS for 14 consecutive days, once daily for 20 minutes. All HD-tDCS interventions in this study were performed with the same instrument at the same location, and the HD-tDCS instrument was operated by the same individual, a graduate student majoring in neurology who has completed professional and standardized training.

### 2.4 Neuropsychological Tests

All participants completed a battery of standardized neuropsychological tests administered by trained neurology graduate students. Emotional status was evaluated using the Generalized Anxiety Disorder-7 (GAD-7) [45] and Patient Health Questionnaire-9 (PHQ-9) [46]. Cognitive function was systematically profiled across domains: global cognition *via* the Montreal Cognitive Assessment (MoCA) [47], language function through the Verbal Fluency Test (VFT) [48] and Boston Naming Test (BNT) [49], memory function using the Auditory Verbal Learning Test (AVLT) [50] (assessing immediate recall, delayed recall, and recognition), and processing speed with the Symbol Digit Modalities Test (SDMT) [51] and Trail Making Test-A (TMT-A) [52]. Executive function was further characterized through attention (Digit Span-forward) [53], working memory (Digit Span-backward) [54], and cognitive flexibility (Trail Making Test-B, TMT-B) [55].

### 2.5 MRI Scans

Structural and resting state-functional MRI (rs-fMRI) for each participant was obtained using a 3-T scanner (Discovery GE750w at Information Science Center of the University of Science and Technology of China). During rs-fMRI scanning, participants were instructed to keep their eyes closed, but not to fall asleep, and to try to think of nothing in particular. Tight but comfortable foam padding was used to minimize head motion, and earplugs were used to reduce scanner noise. The parameters of the different sequences were set as follows:

T2 FLAIR: repetition time (TR)=8,000 ms; time to echo (TE)=165 ms; inversion time (TI)=2,000 ms; flip angle=111°; matrix size=512 × 512; FOV=256 × 256 mm^2^; slice thickness=5 mm, gap=1 mm; total slices=20.

rs-fMRI: TR=2,400 ms; TE=30 ms; flip angle=90°; matrix size=64 × 64; FOV=192 × 192 mm^2^, slice thickness=3 mm, no gap; 46 continuous slices (one voxel=3 × 3 × 3 mm^3^).

Sagittal three-dimensional (3D) T1-weighted images were acquired using a brain volume sequence with 188 slices (TR=8.16 ms; TE=3.18 ms; flip angle=12°; FOV=256 × 256 mm^2^; slice thickness=1 mm, no gap; voxel size=1 × 1 × 1 mm^3^).

### 2.6 fMRI Data Processing

All data were preprocessed using DPASF (Data Processing Assistant for Resting State Functional MR Imaging toolkit) (http://rfmri.org/content/dparsf) [56] and SPM12 (Statistical Parametric Mapping) (https://www.fil.ion.ucl.ac.uk/spm/software/spm12/) based on the MATLAB platform. The main preprocessing steps are as follows: (1) Remove the first five time points to reduce magnetic field instability and adaptation effects; (2) Perform slice-timing correction to eliminate timing differences; (3) Exclude data with excessive head motion(maximum frame-wise displacement > 3 mm) or apply correction to minimize movement effects; (4) Conduct spatial normalization by aligning images with 3D T1 images to register functional data to the Montreal Neurological Institute (MNI) template; (5) Apply a 6mm Gaussian kernel for smoothing; (6) Detrend the data to reduce scanner drift; (7) Use regression to remove white matter, cerebrospinal fluid, and global signals along with head motion parameters; (8) Apply a 0.01-0.1 Hz bandpass filter to suppress high-frequency noise.

### 2.7 Statistical Analysis

All calculations and statistics were implemented in Statistical Package for the Social Sciences (SPSS version 25, IBM Corp., Armonk, NY, USA). The demographic and clinical characteristics across the four groups were compared using appropriate statistical methods. For continuous variables such as age, years of education, neuropsychological test scores, and neuroimaging features of CSVD, comparisons were made using one-way analysis of variance (ANOVA) for normally distributed data or the Kruskal-Wallis rank sum test for non-normally distributed data. Categorical variables, including gender and vascular risk factors, were analyzed using the Chi-square test. For neuropsychological tests and rs-FC strength comparisons before and after the intervention, paired-sample t-tests (for normally distributed data) or Wilcoxon signed-rank tests (for non-normally distributed data) were used. Statistical significance was defined as a two-tailed *P-*value of <0.05.

The rs-FC differences among the four groups were evaluated through one-way ANOVA, based on a gray matter template implemented in DPARSF and SPM12 software. Multiple comparisons were corrected using Gaussian Random Field (GRF) theory, with significance thresholds set at voxel-level *P <* 0.001 and cluster-level *P <* 0.05 (two-tailed).

Correlation analysis between variables was performed using Pearson's correlation for normally distributed data and Spearman's correlation for non-normally distributed data. Statistical significance was determined by a two-tailed *P*-value <0.05, with False Discovery Rate (FDR) correction applied to control for multiple comparisons.

## 3 RESULTS

### 3.1 Demographic Information, Neuroimaging Markers, and Neuropsychological Test Results

A total of 140 participants were included in the first part of the study, including 36 HCs and 104 patients with CSVD. The patients with CSVD were further divided into the mild WMHs group (39 patients), the moderate WMHs group (37 patients), and the severe WMHs group (28 patients).

The results showed no significant differences in age, gender, and years of education among the four groups. In terms of vascular risk factors, the proportion of hypertension in WMH patients is significantly higher than that in the HC group. In addition, the WMH volume, Lacunes, and CMBs in WMH patients were higher, to varying degrees, than in the HC group. The specific differences between groups are presented in Table **1**. However, WMHs patients showed significant differences in MoCA (F = 6.850, *P <* 0.001), AVLT-study (F = 3.981, *P =* 0.010), AVLT-immediate (F = 5.320, *P =* 0.002), AVLT-delay (F = 3.795, *P =* 0.012), AVLT-recognition (F = 13.022, *P =* 0.005), TMT-A (F = 18.679, *P <* 0.001), TMT-B (F = 13.131, *P =* 0.004) and VFT-fruits/vegetables (F = 3.658, *P =* 0.014) compared with HCs showed in Table **2**.

### 3.2 Rs-FC Analysis

Left hippocampal subregions to whole-brain rs-FC analysis showed that the rs-FC strength between HIPc subregion and left dorsolateral superior frontal gyrus and right medial superior frontal gyrus significantly altered in patients with CSVD compared with HCs, as shown in Fig. (**2**).

Further correlation analysis of rs-FC strength and neuropsychological tests showed that the rs-FC strength between HIPc subregion and right medial superior frontal gyrus was negatively correlated with TMT-A in patients with WMHs after false discovery rate correction (r=-0.299, *P*=0.003, corrected *P*=0.033) (Spearman correlation analysis), as shown in Fig. (**3**).

### 3.3 Treatment of HD-tDCS

In the first part of the study, altered rs-FC was found between the hippocampus and the left DLPFC. Meanwhile, considering the important role of the left DLPFC in cognitive function and central electrode selection in previous studies. In the second part of the study, HD-tDCS treatment was performed with the central electrode position at F3 (anodal), and four surrounding electrodes (cathodal at Fp1, Fz, C3, and F7).

A total of 20 participants were included in the second part of the study. The mean age (standard deviation) was 71.15 (7.95) years, and the mean years of education was 13.31 (3.86) years. After 14 days treatment, the participants’ MoCA increased from 18.50 (3.17) to 20.45 (3.73), AVLT-study score increased from 4.71 (1.81) to 6.04 (2.17), AVLT-delay score increased from 3.00 (2.69) to 5.00 (2.98), AVLT- recognition score increased from 11.50 (5.75, 14.00) to 13.00 (9.75, 14.00), SDMT score increased from 14.00 (9.41) to 17.41 (9.66) and DST-backward score increased from 3.00 (3.00, 4.00) to 4.00 (3.00, 5.00) and after treatment, as shown in Table **3**.

### 3.4 ROI-ROI rs-FC Analysis

Four participants were excluded due to excessive head motion during analysis. The rs-FC strength between HIPc and alteration cluster found in the first part of the study, located in the left dorsolateral superior frontal gyrus, was decreased from 0.07 (0.13) to -0.02 (0.10) after treatment, as shown in Fig. (**4**). In further correlation analysis, no significant correlation was observed between changes in rs-FC after treatment and changes in cognitive test scores (MoCA r = −0.13, *P = *0.62; AVLT-study r = −0.12, *P = *0.67; AVLT-delay r = −0.01, *P = *0.98; AVLT-recognition r = −0.15, *P = *0.62; SDMT r = −0.10, *P = *0.73; DST-backward r = 0.43, *P = *0.095).

### 3.5 Adverse Effects

All 20 participants completed the 14-day treatment without any serious adverse events. Only one participant reported mild, transient scalp tingling at the start of stimulation, which resolved shortly after the current stabilized. No seizures, persistent pain, skin burns, or neurological deficits were observed. All the events were tolerable.

## 4 DISCUSSION

In this two-phase study, we first identified disrupted rs-FC within the hippocampal-prefrontal circuit, specifically between the left HIPc and both the left dorsolateral superior frontal gyrus and the right medial superior frontal gyrus in patients with CSVD compared to healthy controls. Afterwards, considering that HD-tDCS is difficult to accurately stimulate the right medial superior frontal gyrus, the left DLPFC was chosen as the stimulation target. Then, we demonstrated that a 2-week HD-tDCS intervention targeting the left DLPFC not only reduces the functional connectivity strength of HIPc-DLPFC but also improves global cognition, memory function, information-processing speed, and attention, providing important evidence for HD-tDCS as a mechanism-guided therapy for patients with CSVD.

Consistent with previous studies [57, 58], this study further confirms that patients with CSVD have significant cognitive impairment, primarily characterized by declines in global cognitive function, memory, executive function, and language function, and that cognitive impairment gradually worsens with disease severity. Among them, the decline in executive function is considered a significant feature of CSVD cognitive impairment, as well as a significant feature of vascular cognitive impairment [58, 59]. The integrity of the prefrontal-hippocampal functional connection plays an important role in cognitive function. More and more researchers have demonstrated that the hippocampus plays an important role in memory and executive function through its connection with the prefrontal cortex [21, 22]. This is consistent with the results of this study, which found that the rs-FC strength between HIPc subregion and left dorsolateral superior frontal gyrus and right medial superior frontal gyrus significantly altered in patients with CSVD. Further correlation analysis showed that the rs-FC strength between HIPc subregion and right medial superior frontal gyrus was negatively correlated with executive dysfunction in patients. In addition, studies have shown that CSVD-induced damage to the white matter pathway is believed to lead to dissociation syndrome [60], resulting in a decrease in the ability to perform complex cognitive tasks that require coordination across multiple cortical regions [61]. Our finding, post-intervention, of improvement in SDMT & DST-forward suggests that HD-tDCS may mitigate this by regulating prefrontal-hippocampal integration, thereby enhancing task-switching efficiency.

While reduced HIPc-DLPFC rs-FC after HD-tDCS may initially appear counterintuitive, this could reflect a normalization of hyperconnectivity driven by pathological compensation. Multiple studies have found increased connectivity between the hippocampus and dorsolateral prefrontal cortex in patients with Alzheimer’s Disease (AD), which researchers believe may be due to increased rs-FC as a compensatory mechanism to resist cognitive impairment in patients with AD [21, 62]. Similarly, in patients with Parkinson's disease, the rs-FC between the hippocampus and prefrontal lobe was significantly also altered in patients with visual hallucinations compared with other patients with Parkinson's disease, and this altered functional connectivity was associated with poorer disease prognosis [22]. In other words, increased rs-FC between the hippocampus and prefrontal lobe may represent inefficient compensatory recruitment of resources to maintain cognitive performance. By enhancing synaptic efficiency through neuromodulation, HD-tDCS may reduce the need for such maladaptive overactivation, thereby improving cognitive metrics while weakening rs-FC strength. This hypothesis aligns with our research findings that after 2 weeks of HD-tDCS treatment, the functional connectivity strength of CSVD decreased, and global cognitive, memory, and executive functions improved. Unfortunately, there was no significant correlation between reduced functional connectivity and improved cognitive function, which may have been overlooked due to insufficient sample size.

The therapeutic potential of HD-tDCS lies in its neuromodulatory mechanisms. By delivering low-intensity current to targeted cortical regions, HD-tDCS directly modulates neuronal excitability and promotes synaptic plasticity within critical cognitive networks [63]. Our findings confirm that this targeted intervention can effectively normalize aberrant functional connectivity and lead to significant improvements in global cognition, memory, and processing speed in patients with CSVD. Crucially, this form of neurophysiological modulation complements the mechanisms of conventional cognitive rehabilitation, which primarily operates through behavioral training and experience-dependent plasticity [64]. Furthermore, growing evidence suggests that combining HD-tDCS with cognitive rehabilitation can yield synergistic effects, thereby enhancing overall therapeutic efficacy [65]. Consequently, integrating these complementary approaches represents a promising strategy for achieving sustained cognitive enhancement, warranting further investigation in future clinical trials.

## STUDY LIMITATION

Although this is an open study with a small sample, it provides new ideas for improving cognitive function in patients with CSVD and is well prepared for our subsequent study. However, this study has notable limitations. Firstly, the small sample size may limit statistical power and generalizability, preventing subgroup analysis. Secondly, as an open-label trial lacking a sham control, placebo effects, particularly for subjective cognitive outcomes, cannot be excluded. Thirdly, the short-term follow-up period (2 weeks) precludes conclusions about the durability of cognitive improvements. Finally, we plan to conduct a multicenter, randomized, sham-controlled trial to increase sample size, recruit participants from diverse racial and geographic backgrounds, and conduct long-term follow-up to confirm the durability of cognitive gains and the generalizability of HD-tDCS in patients with CSVD.

## CONCLUSION

This study suggests HD-tDCS targeting the left DLPFC may improve cognitive function in CSVD by normalizing the rs-FC between the hippocampus and prefrontal lobe. Further validation of the efficacy of HD-tDCS in treating CSVD-related cognitive decline and optimization of stimulation regimens are needed in the future.

## Figures and Tables

**Fig. (1):**
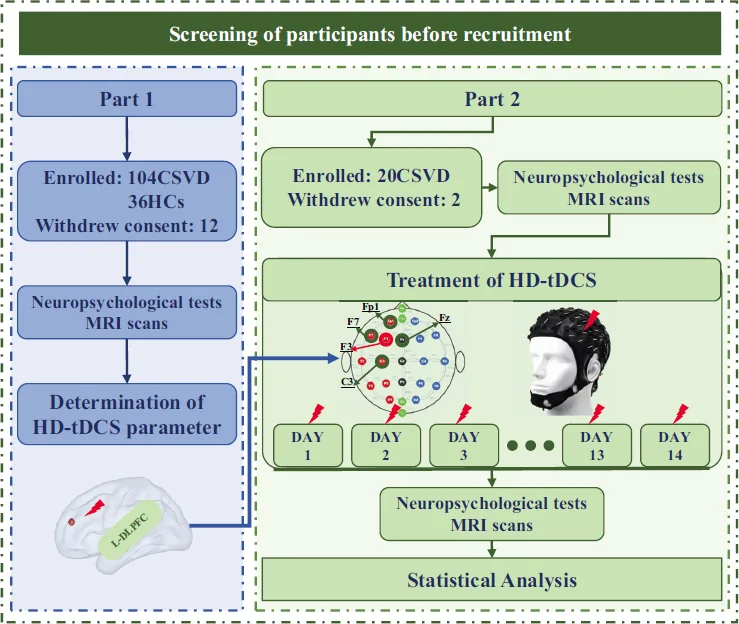
The flowchart of the study. CSVD: cerebral small vessel disease, HD-tDCS: high-definition transcranial direct current stimulation, MRI: magnetic resonance imaging, HCs: Healthy Controls.

**Fig. (2):**
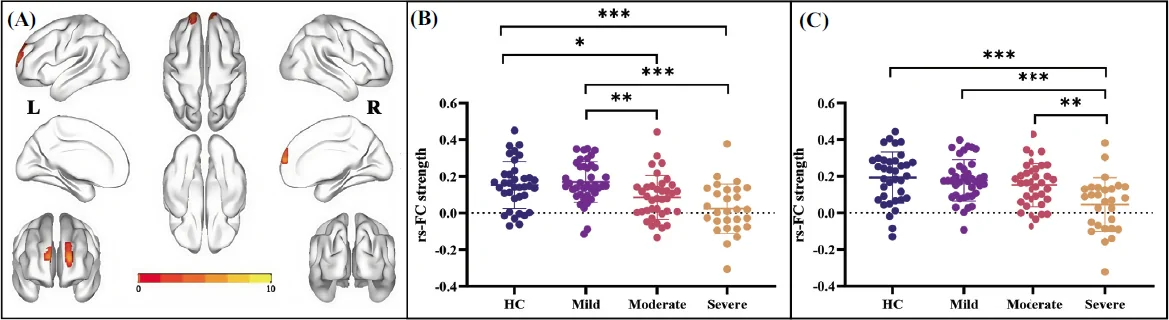
Abnormal rs-FC of the hippocampal-frontal lobe circuit in patients with CSVD. The rs-FC between the HIPc and both the left dorsolateral superior frontal gyrus and the right medial superior frontal gyrus showed significant alterations between the four groups (**A**), and the rs-FC strengths had significant gradient effects in patients with CSVD (**B** and **C**). CSVD: cerebral small vessel disease, rs-FC: resting state functional connectivity, HIPc: hippocampal cognitive subregion.

**Fig. (3):**
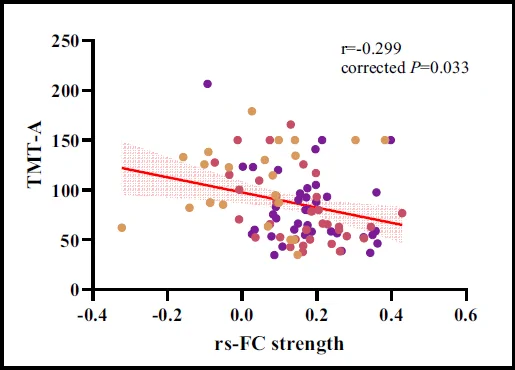
The correlation between the rs-FC strength of the hippocampal-frontal lobe circuit and the TMT-A test in patients with CSVD. The rs-FC strength between the HIPc and the right medial superior frontal gyrus was negatively correlated with TMT-A scores in patients with CSVD. CSVD: cerebral small vessel disease, rs-FC: resting state functional connectivity, TMT: Trail Making Test, HIPc: hippocampal cognitive subregion.

**Fig. (4):**
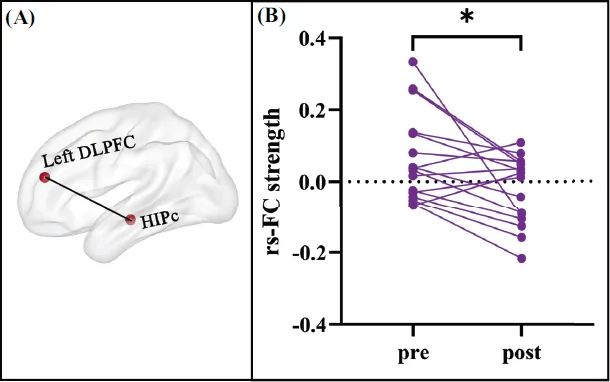
Changes in hippocampal-frontal lobe circuit rs-FC in patients with CSVD after HD-tDCS treatment. (**A**) Schematic diagram of the target circuit: HIPc and left DLPFC. (**B**) Decrease in rs-FC between the HIPc and the left DLPFC after receiving HD-tDCS treatment in patients with CSVD. CSVD: cerebral small vessel disease, HIPc: hippocampal cognitive subregion, DLPFC: dorsolateral prefrontal cortex, rs-FC: resting state functional connectivity, HD-tDCS: high-definition transcranial direct current stimulation.

**Table 1:** Demographics, vascular risk factors, and neuroimaging markers for cerebral small vessel disease in the four groups. (mean (SD) / median (quartile)).

**-**	**HC Group (n=36)**	**Mild WMHs Group (n=39)**	**Moderate WMHs Group (n=37)**	**Severe WMHs Group (n=28)**	**F/H / χ^2^**	***P***
Age (years)^#^	59.00 (56.00, 65.00)	63.00 (57.00, 71.00)	65.00 (56.50, 73.50)	64.50 (61.25, 70.00)	7.593	0.055
Gender (male)^‡^	18	23	23	12	3.011	0.390
Education (years)^†^	9.31 (3.32)	8.41 (4.16)	8.00 (3.84)	7.75 (3.88)	1.083	0.359
Hypertension^‡^	8	21	22	21	19.568	<0.001^***, a,b,c^
Diabetes^‡^	2	8	7	4	3.905	0.272
Hyperlipidemia^‡^	8	10	9	8	0.356	0.949
Smoking history^#^	9	18	15	11	3.808	0.283
Alcohol consumption history^‡^	12	9	16	12	4.346	0.226
Fazekas scores^#^	0.00 (0.00, 0.00)	2.00 (1.00, 2.00)	4.00 (3.00, 4.00)	5.00 (5.00, 6.00)	134.102	<0.001^***, a,b,c,d,e,f^
WMHs volume^#^	/	8353.13 (4934.25, 12916.13)	15771.38 (10575.57, 21984.75)	29948.07 (21135.10, 39985.31)	44.485	<0.001^***, d,e,f^
Lacunes^#^	0.00 (0.00, 0.00)	0.00 (0.00, 1.00)	0.00 (0.00, 1.50)	0.00 (0.00, 1.00)	16.161	0.001^**, a,b,c^
CMBs^#^	0.00 (0.00, 0.50)	0.00 (0.00, 4.00)	1.00 (0.00, 2.00)	1.00 (0.50, 6.00)	17.624	0.001^**, c^

**Note:** SD: standard deviation; WMHs: White matter hyperintensities; CMBs: Cerebral microbleeds; HC: healthy control. ^†^One-way analysis of variance, ^‡^ Chi-squared test, ^#^ using Kruskal-Wallis test; ^***^ significant at 0.001 level, ^**^ significant at 0.01 level, ^*^ significant at 0.05 level (two-tailed); ^a^ HC groups *versus* Mild_WMH groups (*P<*0.05), ^b^ HC groups *versus* Moderate_WMH groups (*P<*0.05), ^c^ HC groups *versus* Severe_WMH groups (*P<*0.05), ^e^ Mild_WMH groups *versus* Severe_WMH groups (*P<*0.05), ^f^ Moderate_WMH groups *versus* Severe_WMH groups (*P<*0.05).

**Table 2:** Neuropsychological tests of participants in the four groups. (mean (SD) / median (quartile)).

**-**	**HC Group (n=36)**	**Mild WMHs Group (n=39)**	**Moderate WMHs Group (n=37)**	**Severe WMHs Group (n=28)**	**F/H**	***P***
MoCA^†^	22.33 (3.09)	21.16 (4.32)	21.06 (3.79)	17.81 (4.91)	6.850	<0.001 ^***^
AVLT-study^†^	8.15 (1.59)	7.77 (2.24)	7.31 (1.52)	6.55 (2.00)	3.981	0.010 ^*^
AVLT-immediate^†^	9.40 (2.30)	8.17 (3.84)	7.88 (2.65)	6.17 (3.27)	5.320	0.002 ^**^
AVLT-delay^†^	9.06 (2.41)	7.95 (3.84)	7.31(2.67)	6.50 (3.13)	3.795	0.012 ^*^
AVLT-recognition^#^	14.00 (13.00, 15.00)	14.00 (12.00, 14.75)	14.00(12.25, 14.75)	12.00 (10.00, 13.75)	13.022	0.005 ^**^
TMT-A^#^	59.33 (46.25, 76.36)	73.45 (55.54, 98.63)	66.09 (52.13, 102.62)	114.74 (82.21, 138.31)	18.679	<0.001 ^***^
TMT-B^#^	119.94 (94.26, 160.93)	139.69 (101.70, 186.00)	136.30 (108.27, 209.43)	185.18 (119.50, 300.00)	13.131	0.004 ^**^
SDMT^†^	25.18 (10.01)	23.00 (11.66)	21.30 (11.10)	17.84 (8.26)	2.098	0.105
DST-forward^#^	7.00 (5.25, 8.00)	6.00 (5.00, 8.00)	7.00 (5.00, 8.00)	6.00 (5.00, 8.00)	3.930	0.269
DST-backward^#^	4.00 (3.00, 5.00)	4.00 (3.00, 5.00)	4.00 (3.00, 4.00)	3.00(2.75, 4.00)	7.054	0.070
VFT-fruits/vegetables^†^	17.26 (3.87)	14.77 (4.47)	15.53 (3.60)	13.67 (5.60)	3.658	0.014 ^*^
PHQ-9^#^	1.00 (1.00, 4.00)	2.50 (0.75, 5.25)	4.00 (2.00, 7.50)	6.00 (2.25, 9.75)	14.396	0.002 ^**^
GAD-7^#^	1.00 (0.00, 4.00)	1.00 (0.00, 4.00)	2.00 (0.00, 5.00)	4.00 (1.25, 7.50)	8.128	0.043 ^*^

**Note:** SD: standard deviation; MoCA: Montreal Cognitive Assessment; AVLT: Auditory Verbal Learning Test; TMT: Trail Making Test; SDMT: Symbol Digit Modalities Test; DST: Digital Span Test; VFT: Verbal Fluency Test. ^†^One-way analysis of variance, ^#^ using Kruskal-Wallis test, ^***^ significant at 0.001 level, ^**^ significant at 0.01 level, ^*^ significant at 0.05 level (two-tailed); ^a^ HC groups *versus* Mild_WMH groups (*P<*0.05), ^b^ HC groups *versus* Moderate_WMH groups (*P<*0.05), ^c^ HC groups *versus* Severe_WMH groups (*P<*0.05), ^e^ Mild_WMH groups *versus* Severe_WMH groups (*P<*0.05), ^f^ Moderate_WMH groups *versus* Severe_WMH groups (*P<*0.05).

**Table 3:** Changes in cognitive function of CSVD patients pre and post HD-tDCS treatment. (mean (SD) / median (quartile)).

**-**	**Pre HD-tDCS Treatment (n=20)**	**Post HD-tDCS Treatment (n=20)**	**t / Z**	***P***	**Effect Sizes (95 % CI)**
MoCA^†^	18.50 (3.17)	20.45 (3.73)	3.746	0.001^**^	0.84(0.32, 1.34)
AVLT-study^†^	4.71 (1.81)	6.04 (2.17)	3.415	0.003^**^	0.78(0.26, 1.29)
AVLT-immediate^†^	3.72 (3.01)	4.67 (3.01)	1.628	0.122	0.38(-0.10, 0.86)
AVLT-delay^†^	3.00 (2.69)	5.00 (2.98)	3.234	0.005^**^	0.78(0.23, 1.32)
AVLT-recognition^#^	11.50 (5.75, 14.00)	13.00 (9.75, 14.00)	2.367	0.018^*^	0.57(0.18, 0.80)
TMT-A^#^	132.92 (104.00, 200.00)	143.27 (104.33, 244.25)	1.288	0.198	0.29(0.01, 0.66)
TMT-B^#^	221.00 (151.55, 300.00)	223.90 (171.64, 300.00)	0.644	0.520	0.14(0.01, 0.60)
SDMT^†^	14.00 (9.41)	17.41 (9.66)	2.312	0.034^*^	0.56(0.04, 1.07)
DST-forward^#^	6.00 (5.00, 8.00)	7.00 (5.25, 8.00)	1.000	0.317	0.21(0.00, 0.61)
DST-backward^#^	3.00 (3.00, 4.00)	4.00 (3.00, 5.00)	2.653	0.008^**^	0.60(0.31, 0.79)
VFT-fruits/vegetables^†^	12.47 (4.93)	12.29 (4.27)	-0.242	0.812	-0.06(-0.53, 0.42)

**Note:** Effect sizes and their 95 % CI were estimated with 5,000 bootstrap percentile resamples, Cohen's d for paired t-test outcomes and Wilcoxon r for Wilcoxon signed-rank outcomes. HD-tDCS: high-definition transcranial direct current stimulation; SD: standard deviation; CI: confidence intervals; MoCA: Montreal Cognitive Assessment; AVLT: Auditory Verbal Learning Test; TMT: Trail Making Test; SDMT: Symbol Digit Modalities Test; DST: Digital Span Test; VFT: Verbal Fluency Test. ^†^using paired t-test, ^#^ using wilcoxon test, ^**^significant at 0.01 level, ^*^ significant at 0.05 level (two-tailed).

## Data Availability

The data that support the findings of this study are available from the corresponding author upon reasonable request.

## LIST OF ABBREVIATIONS

ANOVA: Analysis of Variance
AVLT: Auditory Verbal Learning Test
BNT: Boston Naming Test
CMBs: Cerebral Microbleeds
CSVD: Cerebral Small Vessel Disease
DLPFC: Dorsolateral Prefrontal Cortex
DST: Digit Span Test
FDR: False Discovery Rate
GAD-7: Generalized Anxiety Disorder-7
HC: Healthy Control
HD-tDCS: High-definition Transcranial Direct Current Stimulation
HIPc: Hippocampal Cognitive Subregion
HIPe: Hippocampal Emotional Subregion
HIPp: Hippocampal Perceptual Subregion
MoCA: Montreal Cognitive Assessment
MRI: Magnetic Resonance Imaging
PHQ-9: Patient Health Questionnaire-9
PVSs: Perivascular Spaces
rs-FC: Resting-State Functional Connectivity
SD: Standard Deviation
SDMT: Symbol Digit Modalities Test
TE: Time to Echo
TI: Inversion Time
TMT: Trail Making Test
TR: Repetition Time
VCI: Vascular Cognitive Impairment
VFT: Verbal Fluency Test
WMHs: White Matter Hyperintensities

## STANDARDS OF REPORTING

STROBE guidelines were followed.
